# Carer Engagement and Support in North and West Kent Rehabilitation Services

**DOI:** 10.1192/bjo.2024.562

**Published:** 2024-08-01

**Authors:** Ahmed Elshafei, Martina Heisig, Amanda Fuller

**Affiliations:** KMPT NHS Trust, Maidstone, United Kingdom

## Abstract

**Aims:**

To compare current practice in local Rehabilitation in audit across North and West Rehab Kent units against standards of ‘Triangle of Care’.

Standard 1.3:

Carer's views and knowledge are sought throughout the assessment and treatment process.

Standard 5.2:

An early formal appointment is offered to the carer to hear their story, and history and address the carer's concerns.

Standard 5.10:

The carer is involved in the discharge planning process.

A previous audit was conducted in 2019 using Triangle of Care and AIMS standards. We decided to see whether the standards have been upheld.

**Methods:**

We included all 43 patients admitted over the previous 6-months. No patient had National Opt-Out. The source of information was the RIO system. The data were analysed by 2 investigators.

A data collection form was used:

Question for Standard 1.3: Were the carer's views and knowledge sought throughout the assessment and treatment process? If this was not the case, the reasons were to be specified.

Question for standard Standard 5.2: Was an early formal appointment offered to the carer to hear their story history and address the carer's concerns?

Question for standard Standard 5.10: Was the carer involved in the discharge planning process?

**Results:**

Standard 1.3:

83.72% had contact with a variety of team members throughout their relative's admission. Reasons for non-involvement included lack of consent, unavailable carers, non-attendance, and carer's preference.

Standard 5.2:

Only 60.53% of carers had an early appointment offer, and the expectation that this should occur in 80% of cases was unmet.

Standard 5.10:

(90%) of the patients had carers involved in the discharge planning process, meeting the required standard.

**Conclusion:**

Best Practice:

The audit results demonstrate that carers are involved in their relative's care throughout the admission and discharge process.

Lessons learned:

Compared with the previous audit in 2019, when the criteria for Standard 5.2 were met, carers were offered a formal early meeting significantly less often. Possible reasons could be the pandemic and resulting changes in practice have certainly led to a reduction in face-to-face meetings. Offering individual time to all carers is essential, and efforts should be made to integrate this into practice.

Next steps:

To allocate a team member to offer a meeting with the carer.

To discuss the outcomes with the Carer Champions on each unit, to review what form their support currently takes, and consider how this could link in with the requirements of Standard 5.2.

To re-audit in 1 year.

